# DNMT3L enables accumulation and inheritance of epimutations in transgenic *Drosophila*

**DOI:** 10.1038/srep19572

**Published:** 2016-01-22

**Authors:** Amitava Basu, Archana Tomar, Vasanthi Dasari, Rakesh Kumar Mishra, Sanjeev Khosla

**Affiliations:** 1 Centre for DNA Fingerprinting and Diagnostics (CDFD), Hyderabad, India; 2 Centre for Cellular and Molecular Biology (CCMB), Council of Scientific and Industrial Research (CSIR), Hyderabad, India; 3 Graduate Studies, Manipal University, Manipal, India

## Abstract

DNMT3L is an important epigenetic regulator in mammals, integrating DNA methylation and histone modification based epigenetic circuits. Here we show DNMT3L to be a part of the machinery that enables inheritance of epigenetic modifications from one generation to the next. Ectopic expression of *DNMT3L* in *Drosophila*, which lacks DNMT3L and its normal interacting partners DNMT3A and DNMT3B, lead to nuclear reprogramming that was gradual and progressive, resulting in melanotic tumors that were observed only when these flies were maintained for five generations. This global gene expression misregulation was accompanied by aberrations in the levels of H3K4me_3_ and H3K36me_3_, globally as well as at specific gene promoters. The levels of these epigenetic aberrations (epimutations) also increased progressively across successive generations. The accumulation and inheritance of epimutations across multiple generations recapitulates the important role of DNMT3L in intergenerational epigenetic inheritance in mammals.

For proper functioning of a eukaryotic cell, all the genes must be expressed at their optimum levels. This is achieved by maintaining the epigenetic profile of each genetic loci appropriately made possible by the coordinated cross talk between the various epigenetic components. There are several proteins that can read or write epigenetic modifications in response to cues from other epigenetic modifications associated with a specific locus^1^. Amongst these, DNMT3L is an epigenetic modifier that interacts with de novo methyltransferases DNMT3A/3B and binds to histone H3 in a region that contains H3K4 to bring about DNA methylation^2^,^3^,^4^. It has also been found to be involved in setting up of methylation imprints in mice during gametogenesis^5^,^6^.

Previous work from our laboratory has shown that overexpression of DNMT3L in mammalian cell lines causes nuclear reprogramming^7^. Importantly, the DNMT3L dependent reprogramming was gradual and the morphological phenotypic changes were observed only 20 passages after transfection^7^. Along with finding out the reason for this gradual and progressive nuclear reprogramming we were interested in identifying the mechanism by which DNMT3L achieves it. In particular, we wanted to probe whether the interactions of DNMT3L with DNMT3A/3B on one hand and histone H3 on the other were redundantly influencing a common subset of genes or whether each of these interactions influenced a different subset of genes. The interaction of DNMT3L with histone H3 in the absence of de novo DNA methyltransferases could either be examined in mammals (mice or cell lines) deleted for DNMT3A and 3B or in an organism that normally lacks these proteins. Deletion of DNMT3A and 3B causes lethality in mice^8^,^9^ and cell lines are prone to epigenetic instability^10^. Therefore, we decided to examine these questions in *Drosophila* that lacks DNMT3A/3B as well as DNMT3L. This was important, as it allowed us to probe the interaction of DNMT3L exclusively with histone H3 in transgenic *Drosophila* that ectopically expressed DNMT3L. Moreover, it also allowed us to use the various genetic tools available for this well characterized model organism.

In this study we show that ectopic expression of DNMT3L in *Drosophila* leads to nuclear reprogramming even in the absence of de novo DNA methyltransferases. Similar to our previous observation in mammalian cells, the reprogramming was gradual and progressive with the phenotypic consequences being observed only after maintaining ectopic expression of DNMT3L in *Drosophila* for at least five generations. Reduction in the levels of H3K4me_3_ and H3K36me_3_ along with genome-wide misregulation of genes was progressive, indicating accumulation of altered epigenetic modifications (epimutations) across multiple generations. Interestingly, these epimutations were passed onto the next generation only by the mother, emphasizing the importance of DNMT3L in the establishment of parent-of-origin-specific epigenetic inheritance in mammals.

## Results

### Transgenic *Drosophila* expressing DNMT3L display mild wing phenotype

To understand the functional consequence of DNMT3L interaction with H3, transgenic *Drosophila* carrying the *DNMT3L* transgene (cloned in the pUAST vector under the control of an *hsp70* promoter that contained *GAL4* binding sites [UAS]) was generated by P-element mediated germline transformation (Methods). After injection into *w*^*1118*^ *Drosophila* embryos, 6 independent transgenic *Drosophila* lines with *DNMT3L* (referred to UAS-3L here after) integrated at various loci on different chromosomes were established (Supp Table S1). For constitutive expression of *DNMT3L*, the different UAS-3L transgenic lines were crossed with flies constitutively expressing GAL4 including *Tubulin-GAL4*, *Actin-GAL4* and *daughterless-GAL4*. The progeny *Tubulin-GAL4>DNMT3L*, *Actin-GAL4>DNMT3L* and *daughterless-GAL4>DNMT3L* from these crosses are referred to as *Tub-3L*, *Actin-3L* and *da-3L* respectively in this manuscript. Tissue-specific expression of *DNMT3L* was achieved by crossing these transgenic DNMT3L flies to *GMR-GAL4* (eye-specific) and *hml-GAL4* (Hemolymph-specific) driver stock flies.

**Figure 1:**
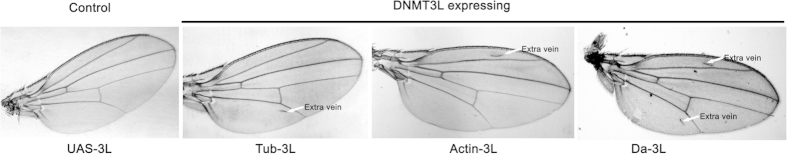
Ectopic *DNMT3L* expression causes wing phenotype in the transgenic *Drosophila*. Comparison of wings from control and transgenic DNMT3L flies showing extra veins in the DNMT3L expressing flies. *Tub-3L*, *Actin-3L*, *da-3L* - transgenic DNMT3L flies with *Tubulin*, *Actin* or *daughterless GAL4* driver respectively. Extra veins in the wings are marked by arrows.

Transgenic flies constitutively expressing *DNMT3L* (*Tub-3L*, *Actin-3L*, *da-3L*) were scored for possible phenotypic defects. In many progeny, phenotypic defect was observed only in the wings. In 50–100% of the progeny, the defect was the appearance of extra vein(s) (Fig. 1, Supp Table S2). The localization of these extra veins differed in individual progenies (Fig. 1). In approximately 2–3% of the progeny, we also observed broken wing phenotype (Supp. Fig. S1).

### Ectopic expression of DNMT3L causes melanotic tumors in *Drosophila* but only in the 5^th^ generation

**Figure 2:**
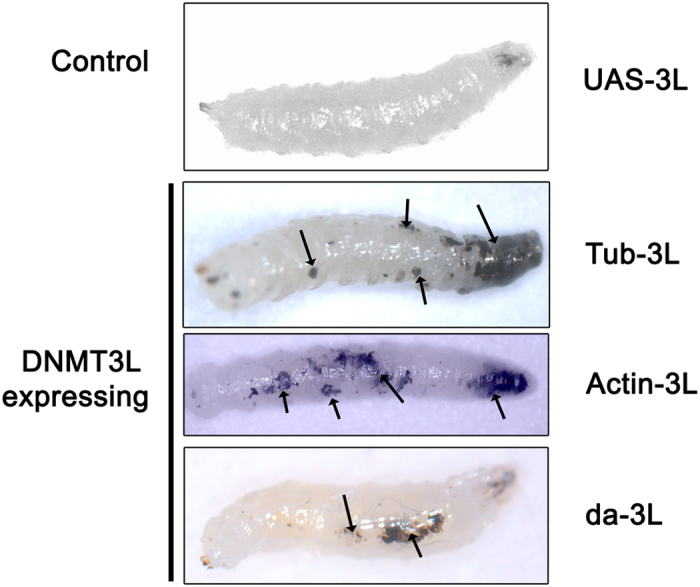
Ectopic *DNMT3L* expression causes melanotic tumors in transgenic flies from the fifth generation. Images of 3^rd^ instar larvae from DNMT3L transgenic flies in the absence and presence of GAL4 drivers. Melanotic tumors were observed in DNMT3L transgenic flies in the larval stage after maintaining the flies for five generations. The melanotic tumors are marked by arrows. The location of the melanotic tumors varied in different larvae. *UAS-3L* - transgenic DNMT3L flies without any *GAL4* driver. *Tub-3L*, *Actin-3L*, *da-3L* - transgenic DNMT3L flies with *Tubulin, Actin* or *daughterless GAL4* driver respectively.

Since we had observed a gradual and cascading nuclear reprogramming over 20 passages in HeLa cell overexpressing *DNMT3L*^7^, we decided to examine the effect of ectopic *DNMT3L* expression in transgenic *Drosophila* maintained across several generations. Homozygous transgenic Tubulin-DNMT3L lines (*Tub-3L*) were bred by self-crossing progeny in each generation. Until the 4^th^ generation (G4), extra veins in the wings or broken wings were the only phenotypes observed as a consequence of the ectopic *DNMT3L* expression. No other morphological abnormalities were observed during any of the embryonic, larval or pupal stages till G4. However, in the 5^th^ generation some of the 3^rd^ instar larvae (~5%) had black masses (Fig. 2, *Tub-3L*) that resembled melanotic tumors^11^. None of these larvae with melanotic tumors survived beyond the larval stages. No pupae or adults flies had melanotic tumors and the remaining progeny developed into normal adults (with several showing the mild wing phenotype), and were fertile. In all the subsequent generations (maintained till G20), 5–8% of the 3^rd^ instar larvae consistently showed melanotic tumors and did not survive whereas the rest of the progeny were normal and fertile (Table 1). Real-Time RT-PCR and Western blot analysis showed that the expression of *DNMT3L* remained constant in all the generations from G1 to G5 (Supp. Figs S2, S3) suggesting that the appearance of the larvae with tumors in 5^th^ generation progeny was not due to an abrupt change in its expression.

It was possible that the appearance of melanotic tumors in the DNMT3L transgenic flies only from the 5^th^ generation was due to (i) an unrelated genetic mutation in these flies; (ii) genomic context of the DNMT3L transgene; or (iii) due to the GAL4 driver line used. To rule out the role of an unlinked genetic mutation in the transgenic flies, the Tubulin GAL4 driver was removed from the transgenic DNMT3L flies after 20 generation (G20) by crossing *Tub-3L* flies to double balancer flies (*Pin/Cyo; Tm*_*2*_*/Tm*_*6*_). No progeny (referred to as UAS-3L* here after) with melanotic tumors was observed in any larvae in subsequent generations (Table 2). This was true for both heterozygous (in G21) and homozygous UAS-3L* (G22 onwards) progeny (Table 2) indicating that the melanotic tumors were due to ectopic expression of *DNMT3L*.

The role of the *GAL4* driver or the genomic context of the DNMT3L transgene in the observed phenotype was ruled out by repeating these crosses with transgenic DNMT3L flies that had DNMT3L transgene integrated at other genomic loci (Suppl Table S1) and crossing them to the constitutive *Actin-GAL4* and *da-GAL4* driver flies. In all the crosses with both *Actin* and *da-GAL4* drivers, progeny with melanotic tumors were again observed only in G5 and subsequent generations (Fig. 2; Tables 3 and 4).

**Figure 3:**
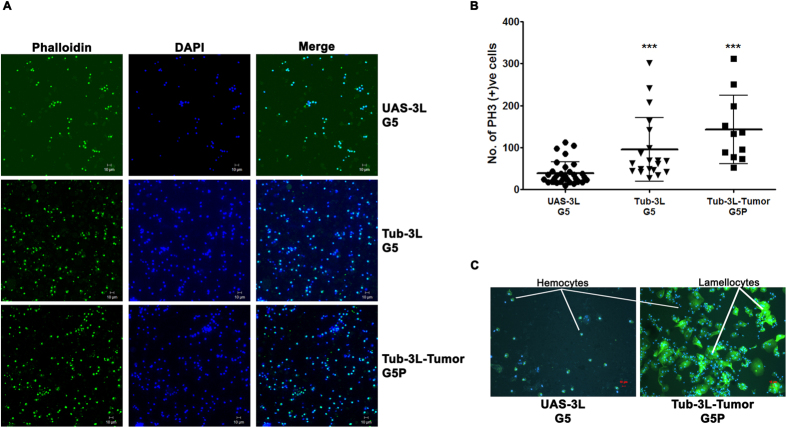
Ectopic *DNMT3L* expression affects the number and types of hemocytes. (**A**) Florescent microscopic images of Alexa 488-Phalloidin (stains Actin) stained hemocytes of control *UAS-3L* and the *DNMT3L* expressing G5, *Tub-3L* transgenic flies. The nuclei of the cells was counterstained with DAPI. Note the increase in the number of hemocytes in the *DNMT3L* expressing *Tub-3L* and *Tub-3L*-tumor transgenic flies. (**B**) PH3 positive (proliferating) cells in control and DNMT3L expressing 3^rd^ instar larvae. (**C**) Hemocytes stained with Phosphohistone 3 (PH3) from 5^th^ generation control and *DNMT3L* expressing larvae (with melanotic tumors). Note the increase in the number of lamellocytes in the larvae bearing the tumors. *UAS-3L*, G5 - 5^th^ generation transgenic *UAS-DNMT3L* flies (without any *GAL4* driver). *Tub-3L*, G5: 5^th^ generation transgenic flies expressing *DNMT3L* with *Tubulin-GAL4* driver. *Tub-3L*-tumor, G5P-: 5^th^ generation transgenic *Tub-3L* flies expressing *DNMT3L* with melanotic tumors. The error bars represent Standard Deviation (S.D.). *Indicate significant difference (Student’s t test, ***p < 0.001).

The melanotic tumors were found to be present in the hemolymph, the circulatory fluid in *Drosophila* that contains free floating hemocytes. To examine the influence of *DNMT3L* expression on the number and types of hemocytes in the hemolymph, the hemolymph from control *UAS-3L* flies was compared with that of 5^th^ generation *Tub-3L* flies with or without melanotic tumors. The number of hemocytes were increased in *Tub-3L* flies (both with and without melanotic tumors; Fig. 3A). The number of proliferating cells as indicated by PH3 antibody staining was also found to be significantly more in G5, *Tub-3L* larvae (Fig. 3B). The types of hemocytes present in the hemolymph was markedly different with the *Tub-3L* flies showing a large numbers of lamellocytes (Fig. 3C).

### Phenotypic defects in transgenic *Drosophila* expressing DNMT3L in a tissue-specific manner also appear in G5

**Figure 4:**
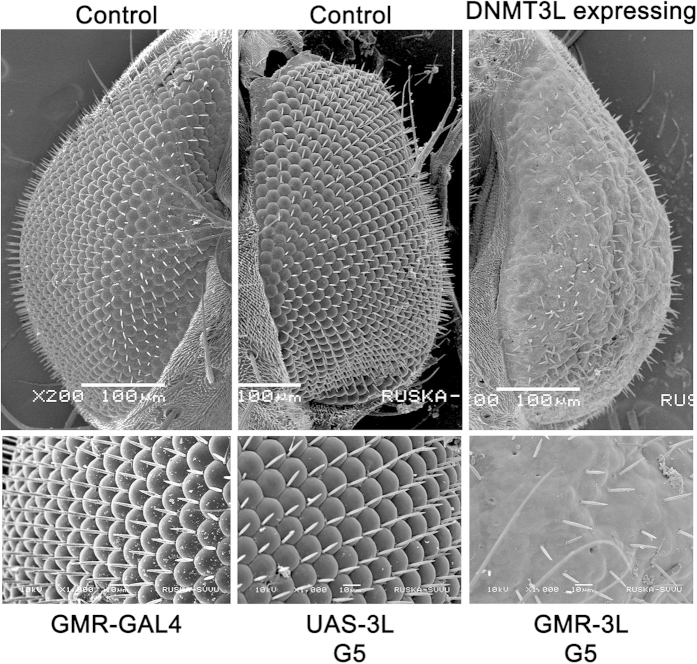
Tissue-specific expression of *DNMT3L* in eyes causes rough eye phenotype in 5^th^ generation flies. (**A**) Scanning electron microscope images of eye from control (*UAS-3L* & *GMR-GAL4*) and *DNMT3L* expressing (*GMR-3L*) transgenic *Drosophila*. Images were captured at 200X in the upper panel. Zoomed images for the same samples were taken at 1000X and are shown in the lower panel. *GMR-GAL4* - Flies containing only the *GMR-GAL4* driver. *UAS-3L*, G5 - 5^th^ generation transgenic *UAS-DNMT3L* flies (without any *GAL4* driver). *GMR-3L*, G5: 5^th^ generation transgenic flies expressing *DNMT3L* with *GMR-GAL4* driver (Eye-specific).

As the melanotic tumors were found to be present in the hemolymph, we wanted to test whether the effect of DNMT3L was only limited to this tissue in *Drosophila*. To investigate this *UAS-3L* flies were crossed with tissue-specific, hemolymph (*Hml*) and eye (*GMR*)-specific, *GAL4* driver flies. No phenotypic abnormality (not even the wing phenotype) was observed in the progeny of these crosses in G1. For *hml-GAL4*>DNMT3L lines (*hml-3L*), 5–7% of the 3^rd^ instar larvae in the 5^th^ and subsequent generations had melanotic tumors as was observed in crosses with the constitutive *GAL4* driver lines (Table 5). No melanotic tumors in the hemolymph were observed in the 3^rd^ instar larvae of flies expressing DNMT3L in the eye (*GMR-GAL4*>DNMT3L and referred to as *GMR-3L*), but all the progeny in the 5^th^ and subsequent generations showed rough eye phenotype (Fig. 4, Table 6). This indicated that the effect of ectopic *DNMT3L* expression was not limited to the hemolymph alone. Though DNMT3L was expressed in eyes of *Tub-3L*, *Actin-3L* or *da-3L* flies, its expression was much lower than in *GMR-3L* line (Supp. Fig. 4). This may be one of the reasons why we did not observe the rough eye phenotype in *Tub-3L*, *Actin-3L* or *da-3L* lines. More importantly, the phenotypic consequence of tissue-specific ectopic *DNMT3L* expression was also manifested only in G5 and later generations, similar to what was seen with constitutive *DNMT3L* expression.

### Ectopic expression of DNMT3L leads to transcriptional misregulation that progressively increases with each generation

**Figure 5:**
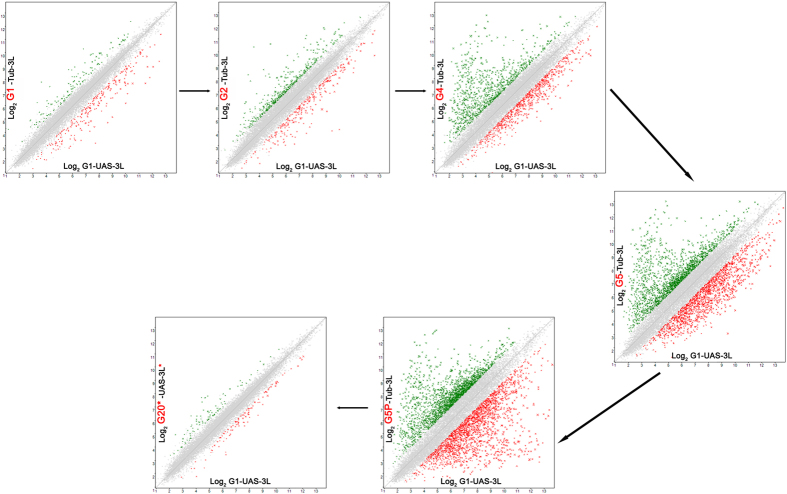
Misregulation of gene expression in flies expressing *DNMT3L* is gradual and progressive. (**A–F**) Scatter plots showing the upregulated and downregulated genes in each generation of the *DNMT3L* expressing transgenic flies. Comparison was made between gene expression levels in 3^rd^ instar *Tub-3L* larvae from a particular generation (indicated on the Y Axis) with *UAS-3L* control larvae from G1. Note a progressive increase in the number of genes that were aberrantly expressed in successive generations. Green crosses represent upregulated genes while red crosses represent down regulated genes. Number of genes misregulated was minimal in G20 flies that lacked *DNMT3L* expression due to the removal of the *GAL4* driver. G1, G2, G4, G5, denotes *Tub-3L* larvae from the particular generation. G5P denotes 5^th^ generation *Tub-3L* larvae with melanotic tumors. G20*-UAS-3L^*^- denotes G20 flies after removal of the *GAL4* driver by crossing flies with double balancers.

To investigate the reasons for the delay in the phenotypic consequences of ectopic expression of *DNMT3L* in *Drosophila*, transcriptional profiling of transgenic DNMT3L larvae from the different generations was performed using Affymetrix *Drosophila* Gene 1.1 ST array (Methods, Data has been submitted to Gene Expression Omnibus (GEO), NCBI, see Supplementary information for details). RNA isolated from third instar larvae (of approximately same stage) of (i) transgenic *Drosophila* expressing DNMT3L in various generations (*Tub-3L*; G1, G2, G4, G5 and G5-phenotype); (ii) control transgenic flies not expressing DNMT3L (*UAS-3L*; G1 & G5); (iii) G20 transgenic flies after removal of *GAL4* driver (UAS-3L*, G20*) (iv) wild type control flies (*w*^*1118*^), were analyzed (see flowchart in Supp. Fig. S5). Expression profiles of larvae in a particular generation was compared to G1 control larvae (*UAS-3L*; G1) to identify misregulated genes by scatter plots (Fig. 5). We noticed a progressive increase in the number of genes that were misregulated upon DNMT3L expression in successive generation. From 205 in G1, the number of misregulated genes progressively increased to 2873 in G5. This number further increased to 3730 in G5 larvae with melanotic tumors (Supp Table S3). It is interesting to note that in all generations subsequent to G1, the number of genes showing repression were relatively more than the overexpressed genes (Supp Table S3). That the observed misregulation was due to the *DNMT3L* expression was reiterated by our finding that the numbers of genes that were misregulated dramatically reduced in G20 larvae (UAS-3L*) after the removal of the *tubulin-GAL4* driver and only 74 genes were aberrantly expressed, most of which did not show altered expression in flies expressing *DNMT3L* in any other generations.

**Figure 6:**
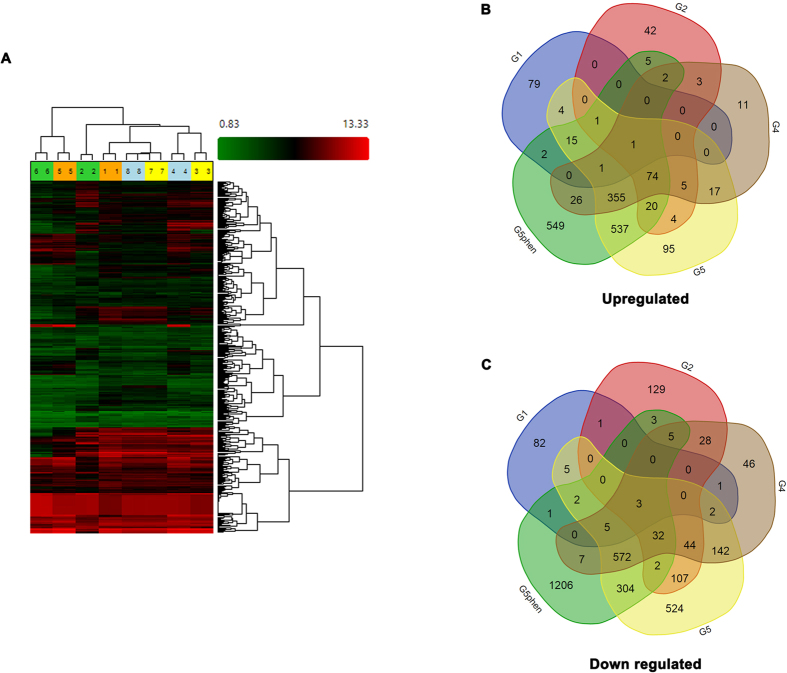
Nuclear reprogramming in DNMT3L expressing *Drosophila*: Microarray data analysis. (**A**) Heat map depicting hierarchical clustering of differentially expressed genes in the various samples and examined by microarray analysis. The heat map shows the relative expression levels of each transcript in each samples. Green denotes downregulated, red denotes upregulated and black depicts genes that showed no change with respect to their expression level in control G1 *UAS-DNMT3L* transgenic larvae. Note the similarity in the heat map profile of the biological replicates for each generation. Sample numbers given above the profile denote: are as follows 1- G1-*UAS-3L*; 2 to 5 –*Tub-3L* larvae from generation G1, G2, G4 and G5; 6 – G5-*Tub-3L* larvae with tumors, 7 – G5-*UAS-3L* and 8- G20*-UAS-3L* larvae from 20^th^ generation where GAL4 driver was removed. (**B**,**C**) Venn diagrams depicting genes upregulated (**B**) and downregulated (**C**) in *DNMT3L* expressing flies across various generations. The number of genes that remain misregulated in successive generations increased progressively (see also Supplementary Table 4). G1 to G5 depict generation number, G5Phen denotes genes misregulated in G5 *Tub-3L* larvae that had melanotic tumors.

Heat map analysis of the gene expression profiles for various generations provided an important clue about DNMT3L function. Comparison of duplicates (RNA isolated from 2 groups of 3^rd^ instar larvae, Fig. 6A) for each generation showed almost the same gene expression profile. That *DNMT3L* expression in *Drosophila* resulted in aberrant expression of the same sets of genes in each generation in each larvae suggested that DNMT3L was acting on a specific set of genes.

Of the 205 genes that showed altered expressed in G1, 103 genes were upregulated and 102 were down regulated. Only 1 out of the 103 upregulated genes maintained higher than normal expression in the subsequent generation. Similarly, only 3 out of 102 downregulated genes remained so in later generations. (Fig. 6B,C). The gene expression of the remaining 201 misregulated genes was either not altered or was misregulated in the opposite direction. From approximately 2% (4 out of 205) in G1 this percentage increased to 32% for the genes that were misregulated in G2 and continued to be similarly misregulated in subsequent generations. The majority of the genes (approximately 81%) aberrantly expressed in G4 continued to be so in G5 and/or in G5 larvae with melanotic tumors indicating a gradual, but progressively increased influence of ectopic *DNMT3L* expression.

**Figure 7:**
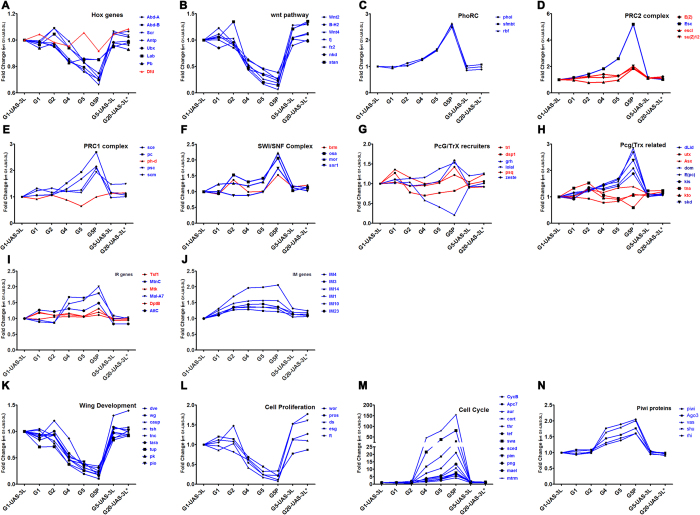
Genes and gene families misregulated across multiple generations in DNMT3L expressing transgenic flies. Graphical representation of gene expression changes (shown as fold change with respect to G1) for the depicted genes across the various generations. Hox gene family (**A**), wnt pathway genes (**B**), Polycomb and Trithorax group of proteins (**C–H**), Immune response (IR) & Immune induced (IM) genes (**I,J**), genes involved in wing development (**K**), cell proliferation (**L**) and cell cycle (**M**) and piwi group of proteins (**N**) were analysed. Y-axis shows fold change with respect to gene expression of the specific gene in control G1 *UAS-3L* (without *Tubulin-GAL4* driver) larvae. G1 and G5-*UAS-3L* are control larvae are without *GAL4* driver from G1 and G5 generation respectively. G1 to G5- *Tub-3L* larvae from the indicated generation. G5P- G5 Tub3L larvae that had melanotic tumors. G20-UAS-3L* denotes larvae from G20 generation after crossing out of the *Tubulin-GAL4* driver. The profile of genes that showed change of gene expression are denoted by blue connecting lines and red connecting line is used to depict genes that do not show a consistent change in gene expression across the various generation. The names of genes examined and their symbols are depicted in the legend for each graph.

Several genes and gene families were affected by ectopic *DNMT3L* expression including the Hox gene family, piwi group of proteins, genes involved in Wnt pathway, wing development, cell cycle, cell proliferation, chromosome organization, Polycomb and Trithorax group of proteins, etc. (Fig. 7). *Drosophila* has 8 *Hox* genes^12^ and in our microarray analysis 7 of them (except *dfd*) were downregulated (Fig. 7A). Similarly, all the 7 *Wnt* genes^13^ were expressed at a significantly lower level after G2 (Fig. 7B). The Polycomb (PcG) and Trithorax (TrX) group of genes are epigenetic modulators of gene expression and include readers and writers of the epigenetic circuitry^14^,^15^. Several members of the PcG/TrX family were found to be misregulated (Fig. 7C–H). Of these, all the PhoRC subfamily members (involved in tethering these complexes to specific DNA sequences) showed a gradual but significant increase in their expression (Fig. 7C). The formation of melanotic tumors in *Drosophila* has been associated with hemocyte-mediated immune response^16^. Several immune response (IR) and immune induced (IM) genes were dysregulated across the various generations (Fig. 7I,J). In addition, the dysregulation of genes involved in wing development, cell cycle and cell proliferation correlated with the phenotypic consequences of *DNMT3L* ectopic expression in *Drosophila* (Fig. 7K–M). Interestingly, all the piwi group of proteins, which are involved in transgenerational epigenetic inheritance^17^, were found to be upregulated (Fig. 7N).

During the analysis of gene expression profiles for individual genes and gene families across different generation we found that the alteration in the expression of most genes was not initiated from G1 but after G2. That the escalation of the aberration at molecular level happened after G2 and not in G1 indicated that a secondary event occurred after 2 generations of ectopic *DNMT3L* expression.

### Epigenetic changes upon ectopic DNMT3L expression are inherited across more than one generation

As DNMT3L has been shown to influence DNA methylation in mammalian cells, we first examined the effect of ectopic *DNMT3L* expression on DNA methylation across successive generations by MeDIP using methyl cytosine antibody. No increase in the level of DNA methylation was detected in any of the generations at the genomic as well as at specific gene promoter level (Supp. Figs S6, S7).

**Figure 8:**
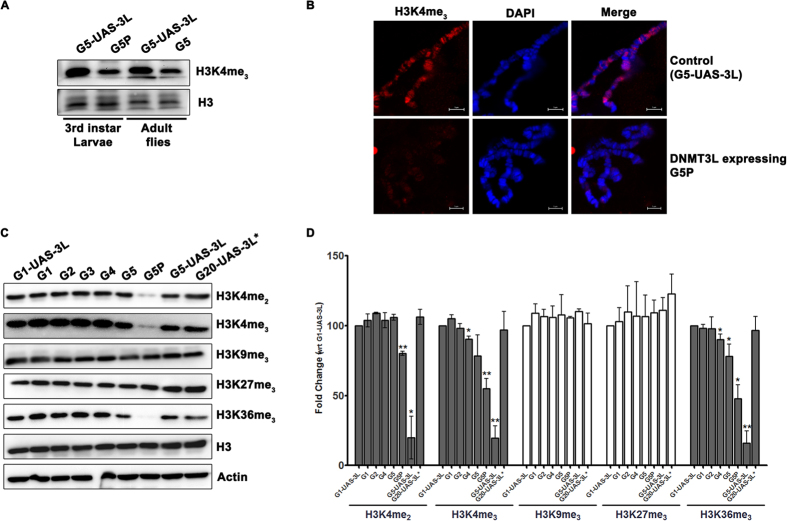
Accumulation of aberrant histone methylation in DNMT3L expressing *Drosophila* across successive generations. (**A**) Western blot analysis demonstrating decrease in the levels of the H3K4me_3_ in 3^rd^ instar larvae or adult flies expressing DNMT3L from the 5^th^ generation as compared to control UAS-3L flies lacking the GAL4 driver. Histone H3 was used as loading control. (**B**) Immunostaining of *Drosophila* polytene chromosomes with H3K4me_3_ antibody. (**C**) Western blot analysis for the various histone modification as indicated, performed on larvae from the various generations of control and DNMT3L expressing *Drosophila* larvae. The densitometric scan values for each signal was calculated and is represented graphically in (**D**) as fold change with respect to the level of the respective histone modification in G1-*UAS-3L* flies lacking *GAL4* driver. G1 and G5-*UAS-3L* are control larvae without *GAL4* driver from G1 and G5 generation respectively. G1 to G5- *Tub-3L* larvae from the indicated generation. G5P- G5 *Tub-3L* larvae that had melanotic tumors. G20-UAS-3L* denotes larvae from G20 generation after crossing out of the *Tubulin-GAL4* driver. Actin was used as a loading control. The error bars represent Standard Deviation (S.D.). *Indicate significant difference (Student’s t test, *p < 0.05, **p < 0.01).

The other known epigenetic partner of DNMT3L is histone H3, particularly when H3 is unmethylated at H3K4^3^. To examine whether ectopic *DNMT3L* expression had any effect on H3 methylation at H3K4, we probed 3^rd^ instar *Drosophila* expressing *DNMT3L* for the level of H3K4me_3_. As can be seen in the representative western blot (Fig. 8A), the level of H3K4me_3_ had significantly reduced in tumor bearing *Tub-3L* (G5) larvae as compared to the control *UAS-3L* (G5) larvae. This observation was reinforced by immuno-staining of polytene chromosome with H3K4me_3_ antibody where negligible H3K4me_3_ staining was observed for the polytene chromosome in *DNMT3L* expressing *Tub-3L* flies (Fig. 8B).

While the phenotypic consequence of *DNMT3L* expression was only observed in G5, the aberrant expression of several genes was observed as early as G2. Western blot analysis was performed to investigate whether the H3K4me_3_ levels were altered in other generations as well. In this analysis, we also tested the levels of other known active (H3K4me_2_, H3K36me_3_) and repressive (H3K9me_3_ and H3K27me_3_) histone marks^18^,^19^. Repressive chromatin marks H3K9me_3_ and H3K27me_3_ were unaffected due to DNMT3L expression but the levels of the active histone marks H3K4me_3_, H3K4me_2_ and H3k36me_3_ were dramatically reduced in tumor bearing G5 3^rd^ instar larvae (Fig. 8C). Furthermore, densitometric scanning of the western blots (Fig. 8D) revealed that the reduction in H3K4me_3_ and H3K36me_3_ was initiated in G3 and the decrease was progressively more in the successive generations with the maximum decrease being observed in tumor bearing 3^rd^ instar larvae in G5. Thus, decrease in the overall levels of active histone modifications in G3 correlated with the escalation of genome-wide aberrant gene expression after G2.

**Figure 9:**
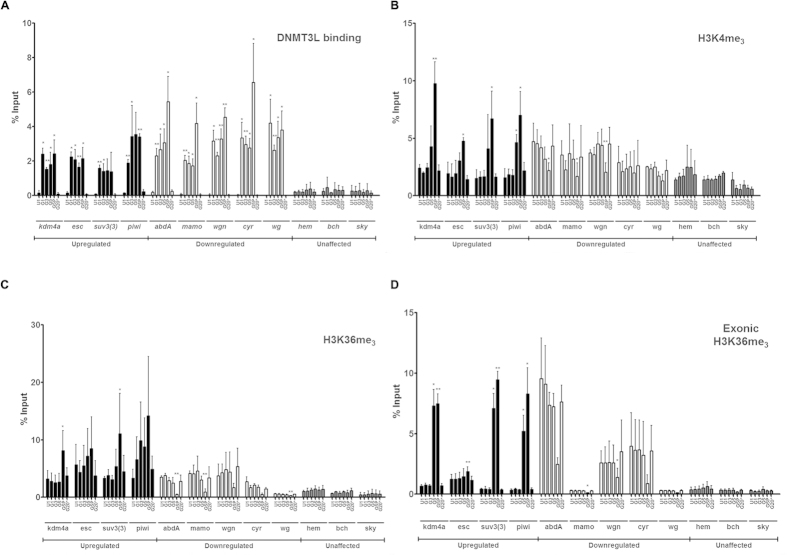
ChIP analysis for association of DNMT3L and specific Histone modifcations with specific gene promoters. Graphical representation of the DNMT3L binding (**A**), association of H3K4me_3_ (**B**) and H3K36me_3_ (**C**) with the promoter and HeK36me_3_ within the exonic regions (**D**) of selected upregulated, downregulated and unaffected genes tested by ChIP using specific antibodies. Results are represented as % input. The names of the specific gene promoters tested are given below the X-axis. U1- control larvae without GAL4 driver from the 1^st^ generation G1, G3 and G5- *Tub-3L* larvae from the indicated generation. G5P denotes 5^th^ generation *Tub-3L* larvae having melanotic tumors. G20* denotes larvae from G20 generation after crossing out of the *Tubulin-GAL4* driver. The error bars represent Standard Deviation (S.D.). *Indicate significant difference (Student’s t test, *p < 0.05, **p < 0.01).

Western blot analysis provided the genome-wide picture of histone modifications profile. To probe the association of histone marks with the promoters of specific genes, chromatin immunoprecipitation (ChIP) was carried out using antibodies to specific histone modifications. The association of DNMT3L with the promoters of these genes was also examined by ChIP. 9 genes that were either upregulated (*kdm4a, piwi, Suv3(3)* and *esc*) or downregulated (*abd-A*, *mamo*, *wgn*, *cyr* and *wg*) were chosen for the analysis. ChIP analysis was also done for 3 other genes (*bch, hem* and *sky*) whose expression was unaffected. DNMT3L was found to be associated with the promoters of all the genes that were aberrantly expressed in *DNMT3L* expressing transgenic flies and in all the generations (Fig. 9A). That this association was genuine was supported by the observation that the promoters of these misreulated genes showed loss of DNMT3L association in the G20 UAS-3L* flies where the Tubulin-GAL4 driver had been crossed out (Fig. 9A). We noticed difference in the profile of DNMT3L binding to the promoters of upregulated and downregulated genes across different generations. While all promoters (for both up and down regulated) showed significant increase in DNMT3L association in G1 *Tub-3L* flies as compared to control UAS-3L flies, the level of DNMT3L associated with the promoters of upregulated genes plateaued in subsequent generations. A slight modification in this trend for upregulated genes was noticed for the *piwi* promoter where further increase in DNMT3L association was observed in G2 followed by plateauing of its association in subsequent generations (Fig. 9A). On the other hand, DNMT3L association with 3 of the 5 down regulated genes promoters was distinctly increased in the tumor bearing G5 larvae as compared to the other generations (Fig. 9A). Amongst the down regulated genes, the association of DNMT3L with the promoter of *abd-A* increased progressively with each successive generation.

For the same set of promoters, ChIP analysis was also carried out using H3K4me_3_, H3K36me_3_ and H3K27me_3_ antibodies (Fig. 9A–C). Since H3K36me_3_ levels in the exonic regions has been associated with regulation of gene expression^20^, we also examined H3K36me_3_ levels within the exonic regions of these genes (Fig. 9D). As we had noted in the western blot analysis, the association of H3K27me_3_ with the various promoters was not altered across the different generations (Supp. Fig. S8) and was in agreement with the microarray data that showed no change in the expression level of *E(Z)*, the H3K27 methyltransferase. At the genome-wide level H3K4me_3_ and H3K36me_3_ levels were drastically decreased in tumor bearing G5 larvae (Fig. 8). Two different trends were noticeable for H3K4me_3_ and H3K36me_3_ (both promoter and exonic region). While the association of H3K4me_3_ and H3K36me_3_ with specific gene promoter was found to have decreased for 3 out 5 the down regulated genes in the tumor bearing G5 larvae (Fig. 9B,C), the promoters of upregulated genes showed an increase in H3K4me_3_ and H3K36me_3_ levels in the tumor bearing G5 larvae. This was also true for the association of H3K36me_3_ with the exonic regions (Fig. 9D). This was a surprising result as association of DNMT3L led to opposite transcriptional outcomes and epigenetic profiles for these two different sets of genes.

DNMT3L is known to be an epigenetic reader and binds to H3 only when H3K4 is in the unmethylated state, a state that is associated with inactive chromatin and transcriptional repression^3^. Our results would indicate that DNMT3L can associate with specific genetic loci even in the presence of H3K4me_3_. Moreover, as the association of DNMT3L with different gene promoters led to changes in the level of H3K4me_3_, it would suggest that DNMT3L binding can also influence the level of H3K4me_3_.

DNMT3L binding within the promoter of the affected genes was observed in G1 and all successive generations but changes in the levels of H3K4me_3_ and H3K36me_3_ was noticeable only from G3. This may suggest that DNMT3L likely influences H3K4me_3_ and H3K36me_3_ indirectly by modulating the expression of H3K4me_3_ and H3K36me_3_ specific histone methyltransferases or demethylases. This contention was backed by our observation that the change in the expression of histone H3K4 and H3K36 methyltransferases and demethylases, especially *Su(var)3-3* and *Kdm4a*, mirrored the decrease in the levels of H3K4me_3_ and H3K36me_3_ (Supp. Fig. S9).

The modENCODE database^21^ for *Drosophila* has catalogued histone modifications associated with specific genetic loci within the *Drosophila* genome for the various adult tissue of the adult flies as well as for embryo and larvae at different developmental stages (Comprehensive encyclopedia of genomic functional elements in the model organisms *C. elegans* and *D. melanogaster*; http://www.modencode.org/; date of access, 15/9/15). Using the data available for 3^rd^ instar larvae in the modENCODE database, we extracted the normal epigenetic profile of the genes that were found to be misregulated in *DNMT3L* expressing flies across the various generations. The epigenetic profile of a region 1000 bp on either side of the Transcriptional Start Site (TSS) for each gene was captured and used for the analysis. Of the 205 genes (103 up and 102 down regulated) misregulated in G1, 153 (~75%) were unmethylated at H3K4. For the complete set of *Drosophila* genes that were represented on the microarray, only 46% of the gene promoters were unmethylated at H3K4. This suggested that in G1, DNMT3L was targeting gene promoters devoid of H3K4me_3_. However, in successive generations this bias was progressively removed (Table 7). In fact, DNMT3L binding was found to be biased for H3K4me_3_ promoters in tumor bearing G5 larvae as 63% of the misregulated gene promoters were found to be trimethylated at H3K4 (Table 7). When the same analysis was performed on misregulated genes after categorizing them as up or down regulated, it was noticed that while both sets showed bias for unmethylated H3K4 in G1, the bias favoring H3K4me_3_ in the following generation was only seen in the down regulated genes. For the upregulated genes the bias for unmethylated H3K4 was removed progressively and by G5 almost equal number of genes were associated or devoid of H3K4me_3_ (Table 7).

### Inheritance of epimutations between two generations in DNMT3L expressing *Drosophila* is dependent upon *piwi*

The progressive decrease in H3K4me_3_ and H3K36me_3_ indicated that alteration in epigenetic modifications (epimutations) were being inherited from one generation to another. Recent studies have implicated the role of *piwi* associated piRNAs in transgenerational inheritance of epigenetic characters^22^,^23^. Examination of the microarray data indicated that all the members of the *piwi* group of proteins were upregulated in *DNMT3L* expressing flies (Fig. 7N). To investigate the role of piwi protein, G5, *Actin-3L* flies were crossed with piwi mutant flies (*piwi*^*06843cn1/CyO*^*; ry*^*506*^) and the progeny of this cross (*piwi*-*Tub-3L*) were scored for melanotic tumors. As shown in Table 8, *piwi*-*Actin-3L* flies carrying mutated *piwi* gene and DNMT3L transgene did not produce any progeny that had melanotic tumors implicating piwi protein in the inheritance of epimutations. Expression of DNMT3L was unchanged in the *piwi*-*Tub-3L* flies and similar to what was observed in G5, *Tub-3L* flies.

### Maternal transmission of epimutations

We next investigated whether the epimutations were being inherited through both the germ lines or were being passed on to the progeny by one parent. The phenotypic consequences of ectopic DNMT3L expression in *Drosophila* appeared only in the 5^th^ generation even though the epimutations (loss of H3K4me_3_ and H3K36me_3_) were observed as early as the 3^rd^ generation. Moreover, the extent of these epimutations increased progressively in subsequent generations. Appearance of tumors only in the 5^th^ generation and in each generation thereafter would indicate that from the 5^th^ generation onwards a threshold level of epimutations necessary for the phenotype accumulates and inheritance of which causes tumors in the progeny. If the threshold level of the epimutations was achieved only in G5 and were being passed on to the progeny, then in a cross between G5 *Tub-3L* and G1 *Tub-3L* flies, some of the progeny should show tumors. Based on this contention, the following crosses were set up: (i) G5-*Tub-3L* ♀ X G1-*Tub-3L* ♂– to check for maternal transmission; (ii) G1-*Tub-3L* ♀ X G5-*Tub-3L* ♂ – to check for paternal transmission; (iii) G1-*Tub-3L* ♀ X G1-*Tub-3L* ♂– negative control; (iv) G5-*Tub-3L* ♀ X G5-*Tub-3L* ♂ – positive control. The results from these crosses are presented in Table 9. As expected G5-*Tub-3L* ♀ X G5-*Tub-3L* ♂ produced 5–6.9% larvae that had melanotic tumors. None of the 3^rd^ instar larvae from the crosses G1-*Tub-3L* ♀ X G1-*Tub-3L* ♂ had tumors. Progeny from G1-*Tub-3L* ♀ X G5-*Tub-3L* ♂ did not show tumors but 2.9–3.7% of the progeny from the G5-*Tub-3L* ♀ X G1-*Tub-3L* ♂ developed melanotic tumors indicating maternal inheritance of the epimutations.

## Discussion

The inheritance of epigenetic modifications across multiple generations results from events during germ cell development and fertilization that allow epigenetic marks acquired during the life time of an organism to be passed on to the progeny^24^. Epigenetic inheritance could be intergenerational as has been documented for genomic imprinting^25^ or transgenerational where epigenetic modification acquired in one generation is inherited unchanged across several generations^26^,^27^,^28^. In both cases, limited knowledge is available on the components and mechanism by which epigenetic modifications cross the meiotic barrier between two generations^29^. A few recent studies have found piwi group of proteins and piRNA to be involved^22^,^23^, however the exact mechanism(s) still needs to be dissected out. Our observation that ectopic expression of DNMT3L in *Drosophila* resulted in initiation and maternal inheritance of epimutations across several generations not only establishes the role of DNMT3L in intergenerational epigenetic inheritance, but also provides a reason to reevaluate the theories that discuss the need for genomic imprinting in placental mammals.

### DNMT3L facilitates nuclear reprogramming even in the absence of de novo DNA methyltransferase

The functional consequence of DNMT3L action is mediated by its interactions with de novo methyltransferases DNMT3A and DNMT3B and histone H3^2^,^3^,^4^. Previous work from our laboratory had indicated a role of DNMT3L in nuclear reprogramming in mammalian cell lines^7^. In the present study, ectopic DNMT3L expression in *Drosophila* (DNMT3L is not present in the *Drosophila* genome) caused progressive misregulation of genes and by the 5^th^ generation a very large number of genes (3730) were aberrantly expressed. Some of the transgenic flies expressing DNMT3L also developed melanotic tumors and did not survive. *Drosophila* has negligible DNA methylation^30^ and lacks the de novo DNA methyltransferases DNMT3A and DNMT3B as well as the maintenance DNA methyltransferase DNMT1. While the methyltransferase, DNMT2, is present in *Drosophila*, it has been shown to be a tRNA methyltransferase^31^. Our results show no change in DNA methylation (globally as well as at specific gene level) in any of the generations indicating that even in the absence of an endogenous functional DNA methyltransferase, the interaction of DNMT3L with histone H3 is sufficient for its nuclear reprogramming capability. It would also suggest that the interaction of DNMT3L with DNMT3A and DNMT3B versus the binding of DNMT3L with histone H3 may be redundant and it would be of interest to determine whether or not both events would influence the same subset of genes.

### DNMT3L facilitates intergenerational inheritance of epimutations

Nuclear reprogramming observed in DNMT3L expressing *Drosophila* (this study) and mammalian cells overexpressing DNMT3L^7^ was gradual and progressive. However, an important difference exists between the two results. While the nuclear reprogramming in mammalian cells occurred across several mitotic divisions^7^, the progressive misregulation of genes across several generations in transgenic *Drosophila* involved germline passage through meiotic divisions. Most epigenetic marks are normally erased and reset in the germline though some studies have shown transgenerational germline passage of epigenetic marks^32^,^33^,^34^,^35^,^36^,^37^. The inheritance of aberrant epigenetic modifications or epimutations across several generations that we observed in this study would fit into the broad definition of transgenerational epigenetic inheritance. However, an important criterion for categorizing inheritance of epigenetic marks as transgenerational is the ability of epigenetic marks to survive germline passage across several generations in the absence of the causative environmental or genetic cue in the subsequent generations^32^. In this study, inheritance of epimutations across successive generation was observed, but only when the causal DNMT3L expression was present. In G20, when the *GAL4* driver was removed from the transgenic flies, the aberrant expression of genes and the epimutations disappeared in the absence of DNMT3L expression. This would indicate that the inheritance of epimutations was intergenerational rather than transgenerational. An important example of intergenerational inheritance is genomic imprinting and DNMT3L has been linked to setting up of methylation imprints in the germ line of mammals (mice)^5^,^6^. Genomic imprinting represents a phenomenon wherein epigenetic modifications are inherited to the progeny in a parent-of-origin-specific manner^38^. In transgenic *Drosophila*, epimutations were inherited from the mother to her progeny suggesting of a possibility that expression of *DNMT3L* had allowed a process similar to genomic imprinting to be initiated. Like in mammals, the formation of germ cells in *Drosophila*, takes place at a time during embryogenesis when the embryo is still under the influence of maternal factors^39^. Therefore, the possibility also exists that the inheritance of epimutations were due to the influence of maternally produced DNMT3L. While further work is needed to distinguish the two possibilities, a closer examination of our result suggests that the possibility of a process similar to genomic imprinting cannot be ruled out. From the 5^th^ generation onwards, 5–8% of the *DNMT3L* expressing larvae showed melanotic tumors. However, in our cross where G5 females with G1 males only 3–4% of progeny showed tumors (Table 9). If only the epimutations passed on through the female germline were responsible for the phenotype then in this cross the percentage of larvae showing melanotic tumors should have remained in the 5–8% range. Decrease in this percentage could suggest that some epimutations at specific genomic loci may be being passed on through the paternal germline. Since we were looking only at the gross phenotypic levels examination of the intergenerational inheritance of epimutations at specific loci would help in distinguishing between the two possibilities.

### DNMT3L allows accumulation of epimutations across more than one generation

As mentioned earlier, established epigenetic modifications are normally reset in the germ cells before fertilization^31^. Therefore, any epimutation acquired during one generation would be lost in the germ cells and hence not inherited. Even for imprinted genes, the epigenetic imprints inherited from the previous generation are erased before new epigenetic modifications determined by the sex of the individual are initiated. However, we observed a progressive decrease in the genome-wide levels of H3K4me_3_ and H3K36me_3_ across more than one generation. This was also true at specific gene levels. For example, the level of H3K4me_3_ at the promoter of the *hox* gene, *abd-A*, decreased progressively across successive generations. Similar inheritance of reduced H3K4 methylation across several generations in mice was recently reported^40^. The accumulated decrease in these modifications (or accumulation of epimutations) suggested that in the presence of DNMT3L new epigenetic modifications were being added over and above of what was inherited from the previous generation. Previous studies on DNMT3L have suggested its role in initiating or setting up of methylation imprints in mammalian germ cells^5^. Based on this, one possible explanation for epimutation accumulation could be incapacitation of protein(s) responsible for erasure or resetting of epigenetic modification, as a secondary event in *Drosophila* expressing DNMT3L. Hence, in each generation DNMT3L would keep adding new epimutations to the ones inherited from the previous generation without being erased. On the other hand, it is possible that the presence of DNMT3L prevented erasure of epimutations inherited from the previous generation. In this scenario, epigenetic modifiers responsible for setting up of new epigenetic marks in the germ cells would add on to the epigenetic modifications that were not erased due to the presence of DNMT3L. Careful examination of the temporal expression of DNMT3L in mammalian germ cells or derivation of transgenic *Drosophila* expressing DNMT3L in a very narrow developmental window immediately before or after the time when epigenetic modifications are reset in the germline would help in answering this question. Moreover, further work to understand, (i) how epimutations were inherited in *hml-3L* and *GMR-3L* flies where DNMT3L expression was restricted to hemolymph and eyes respectively; and (ii) how epimutations that were accumulated over multiple generations reverted back immediately once the ectopic expression of the *DNMT3L* transgene was abolished, would help in understanding the mechanism underlying *DNMT3L* action.

Whether it is in placental mammals, angiosperms or a few species of insects, the phenomenon of genomic imprinting has been invoked as a means to regulate the parasitic relationship that the embryo has with its mother^41^. If the possibility that DNMT3L allowed parent-of-origin-specific intergenerational inheritance of epigenetic modifications, was found to be true then it would be prudent to reevaluate the reason as to why some species have chosen genomic imprinting as a means of regulating gene expression.

## Methods

### Fly stocks and crosses

DNMT3L cDNA with the FLAG tag was PCR amplified from pcDNA3.1-DNMT3L^7^ and sub-cloned into the *Drosophila* cloning vector pUAST under the UAS containing *hsp70* promoter. This vector also contains the reporter *mini-white* gene for the selection of the transgenic lines. Transgenic flies, using the *Drosophila* strain *W*^*1118*^, were generated by standard protocol involving P-element mediated germline transformation^42^. After injection, 6 independent transgenic lines, with integration of DNMT3L transgene on various chromosomes, were established. The induction of the DNMT3L in different lines (*UAS-FLAG-DNMT3L*) was carried out by crossing these lines with constitutive *GAL4* driver lines like *Tubulin-GAL4*, *Actin-GAL4*, and *daughterless-GAL4* or with tissue specific *GAL4* driver like *GMR-GAL4* (eye-specific) and *hml-GAL4* (haemolymph-specific). All *GAL4* driver and the *piwi* mutant (*piwi*^*06843cn1/CyO*^*; ry*^*506*^) lines were obtained from the Bloomington stock center.

### Crosses to get transgenic flies expressing DNMT3L

Initially, the *UAS-DNMT3L* flies and the *GAL4* driver flies were crossed to the double balancer flies to get the desired marker on each chromosomes. The desired flies from both these crosses were further crossed with each other to bring *DNMT3L* and the *GAL4* driver into the same flies. The resulting flies were then self-crossed with each other to obtain homozygous DNMT3L flies with the appropriate GAL4 driver. In case of the crosses with *Tubulin-GAL4* and *da-GAL4* driver flies (*Tubulin-GAL4* and *da-GAL4* driver are present on the 3rd chromosome), the DNMT3L line 38.1.1 in which the *DNMT3L* transgene was on the second chromosome was used. For *Actin-GAL4* and *hml-GAL4* (*Actin-GAL4* and *hml-GAL4* driver on 2^nd^ chromosome, the DNMT3L line 42.1.2 with the transgene on the third chromosome was used. For crosses with *GMR-GAL4* (*GMR* driver present on the 1^st^ chromosome), the DNMT3L line 38.1.1 in which the DNMT3L transgene was on the second chromosome was used.

### Phenotypic Analysis

#### *Drosophila* wing mounting

Wings were removed from both control and transgenic DNMT3L flies and incubated in 10% KOH at 70 °C for 5 minutes followed by washing with PBS. The treated wings were mounted in Canada balsam on a glass slide and photographed using Zeiss Axiocam camera.

#### Imaging of *Drosophila* eyes by Scanning Electron Microscope (SEM)

*Drosophila* heads for imaging were fixed in 2.5% Glutaraldehyde in 0.1 M phosphate buffer (pH 7.2) for 24 hours followed by incubation in 2% aqueous osmium tetraoxide for 4 hours. The samples were serially dehydrated in alcohol and dried to critical point drying with CPD unit. The processed samples were mounted on the stubs with double sided carbon conductivity tape and coated with gold in an automated sputter coater for 3 minutes. Images were acquired using a Scanning Electron Microscope (SEM- Model JOEL-JSM 5600) at 200X or 1000X at RUSKA Laboratory, College of Veterinary Sciences, Hyderabad, India.

### Hemolymph analysis

Third instar larvae were washed with PBS followed by 70% ethanol and blotted dry. The larvae were dissected by gently pulling the epidermis apart with the help of forceps to ooze out the hemolymph on a glass slide. The hemolymph was air dried and further processed. For Phalloidin staining of the hemolymph the cells in the dried hemolymph were fixed in 4% paraformaldehyde for 10 minutes. Excess paraformaldehyde was removed from cells by giving three PBST (PBS with 0.1% triton X-100) washes. A 1:500 dilution of Alexa 488 conjugated anti-Phalloidin antibody (Life Technologies) was added to the sample, the slide was incubated in a humidified chamber for 30 minutes followed by three PBST washes. The slides were mounted with DAPI and observed under florescent microscope. For phospho-histone 3 staining of the hemocytes, 1:100 dilution of mouse anti-PH3 antibody (Millipore) and 1:250 dilution of anti-mouse Alexa Flour 488 (Life Technologies) were used for the immunostaining.

### Chromatin immunoprecipitation (*ChIP)*

Chromatin immunoprecipitation was performed as described previously^43^. Briefly, 3^rd^ instar larvae was homogenised and then sonicated in Biorupter (Diagenode) biorupter so that DNA was sheared between 200–500 bp. Chromatin immunoprecipitation was performed on the sonicated sample using auto ChIP kit (Diagenode) in the IPStar automated ChIP machine (Diagenode) using specific antibodies.

### Antibodies and primers used for the study

The Flag antibody was purchase from Sigma (F1804). H3 (ab1791), H3k4me_3_ (ab8580), H3K9me_3_ (ab8898), H3K27me3 (ab6002), H3K36me_3_ (ab9050) and beta-actin (ab8227) antibodies were purchased from Abcam. Oligonucleotides used in this study are provided as supplementary data.

### Polytene chromosome immunostaining

Immunostaining of the polytene chromosome from control and the transgenic larvae was done following established protocol^44^. Incubation with the primary antibodies against Flag tag (Sigma), histone H3 and H3K4me_3_ (Abcam) was done overnight at 4 °C.

### Microarray analysis

#### Isolation of RNA from *Drosophila*

The RNA for the microarray analysis was isolated from third instar larvae using TRI reagent (Sigma). The RNA was further purified using purelink RNA mini kit (Life Technologies). The RNA for the various samples and their duplicates was from 20–30 larvae at approximately same stage of 3^rd^ instar larval development. The total RNA was analysed for gene expression profiling using the Affymetrix *Drosophila* Gene 1.1 ST array (Affymetrix, Santa Clara, CA) at Imperial Life Sciences, India. This array contained 362,078 probe sets representing more than 15,309 transcripts, including all known genes. Synthesis and labelling of complementary DNA targets, hybridization and scanning of GeneChip were carried out according to the instructions provided by Affymetrix.

#### Bioinformatic analysis

For initial gene level expression analysis, the Affymetrix CEL-files were first imported into Affymetrix Expression Console Software, version 1.3. The Robust Multichip Analysis (RMA) algorithm was applied to the probe cell intensity data files for all experimental conditions, using default parameters in the RMA-sketch workflow for core gene level analysis. Analysis of differentially expressed genes was performed with Affymetrix Transcriptome Analysis Console (TAC) Software. Significance of the difference for each gene was determined by one-way ANOVA. Genes that were differentially regulated by 2 fold or greater with a p-values ≤ 0.05 were included in the further analysis. The scatter plot for the various upregulated and downregulated genes for the comparison between the transgenic flies without *GAL4* driver and the transgenic flies of various generation with *Tubulin-GAL4* driver was made using the Transcriptome Analysis Console (TAC) 2.0 software. Pathway analysis was done using DAVID Bioinformatics Resources 6.7^45^ and Gorilla^46^. The Venn diagrams were created using the web tools provided in the Bioinformatics and Evolutionary Genomics facility website (Van de Peer Y. *et al*. Calculate and draw custom Venn diagrams; http://bioinformatics.psb.ugent.be/webtools/Venn/; date of acess, 15/9/15).

modENCODE (Comprehensive encyclopedia of genomic functional elements in the model organisms *C. elegans* and *D. melanogaster*; http://www.modencode.org/, date of access, 15/9/15) database for *Drosophila melanogaster* was searched for the availability of experiments for H3K27me_3_, H3K36me_3_, H3K4me_2_ and H3K4me_3_ histone modifications in 3^rd^ instar larval stage. A total of 89 dataset were found relevant and downloaded for further comparisons. The coordinates for 1000 bases upstream of TSS and 1000 bases downstream of TSS of up and down regulated genes identified using microarray data genes were calculated. BEDtools was used to identify regions that showed presence of a particular modification in at least 50% of the data sets and designated as regions associated with the specific histone modification whereas all other regions were taken be regions lacking that modification.

For validation of the array data, cDNA synthesis was performed on DNase treated RNA and qRT-PCR (ABI 7500) was set up for the indicated misregulated genes.

## Additional Information

**How to cite this article**: Basu, A. *et al*. DNMT3L enables accumulation and inheritance of epimutations in transgenic *Drosophila*. *Sci. Rep.* **6**, 19572; doi: 10.1038/srep19572 (2016).

## Supplementary Material

Supplementary Information

## Figures and Tables

**Table 1:** Number of larvae with melanotic tumors observed in *Tubulin-GAL4>DNMT3L* (*DNMT3L* expressing flies), across various generations.

Generation No.	Total larvaescreened	Larvae withtumor	% of larvaewith tumor
G5	103	6	5.8
G6	108	7	6.4
G7	118	7	5.9
G8	141	9	6.3
G9	130	7	5.3
G10	114	7	6.1
G11	153	8	5.2
G12	111	6	5.4
G13	128	10	7.8
G14	137	7	5.1
G15	125	6	4.8
G16	141	7	4.96
G17	134	8	5.97
G18	121	6	4.95
G19	145	9	6.2
G20	127	6	4.72

**Table 2:** Melanotic tumors are not observed after the removal of *Tubulin*-GAL4 driver.

Generation No.	Crosses	No. of larvae with tumors/TotalNo. of Larvae		
		Set1	Set2	Set3
G21	G20(*Tub-3L*) ♀ X db ♂	0/118	0/116	0/108
	db♀ X G20(*Tub-3L*) ♂	0/116	0/112	0/121
G22	G21 (UAS-3L^*^) ♀ X db ♂	0/111	0/96	0/101
	db ♀X G21(UAS-3L^*^) ♂	0/98	0/106	0/110
G23	G22 (UAS-3L^*^)♀ X db ♂	0/101	0/102	0/95
	db ♀X G22(UAS-3L^*^) ♂	0/103	0/105	0/97
G24	G23(UAS-3L^*^)♀ X db ♂	0/103	0/107	0/109
	db ♀ X G23(UAS-3L^*^) ♂	0/122	0/110	0/113
G25	G24(UAS-3L^*^)♀ X db ♂	0/108	0/98	0/101
	db ♀ X G24(UAS-3L^*^) ♂	0/107	0/89	0/104

*Tub-3L*- DNMT3L expressing flies with *Tubulin-GAL4* driver; db- Double balancer flies (*Pin/CyO; Tm*_*2*_*/Tm*_*6*_); UAS-3L^*^ – DNMT3L flies after removal of the *Tubulin-GAL4* driver.

**Table 3:** Number of larvae with melanotic tumors observed in *Actin-GAL4>DNMT3L* flies across various generations.

Generation No.	Total larvaescreened	Larvae withtumor	% of larvaewith tumor
G5	119	7	5.8
G6	119	7	5.8
G7	97	5	5.15
G8	107	6	5.6
G9	121	8	6.6
G10	120	6	5
G11	110	6	5.45
G12	122	6	4.9
G13	113	6	5.3
G14	115	5	4.34
G15	95	5	5.2
G16	132	7	5.3
G17	96	5	5.2
G18	121	7	5.78
G19	111	7	6.3
G20	116	7	6.03

**Table 4:** Number of larvae with melanotic tumors observed in *da-GAL4>DNMT3L* flies across various generations.

Generation No.	Total larvaescreened	Larvae withtumor	% of larvaewith tumor
G5	116	6	5.17
G6	102	5	4.9
G7	100	6	6
G8	89	4	4.49
G9	115	7	6.08
G10	97	4	4.12
G11	121	8	6.61
G12	121	7	5.78
G13	128	5	3.9
G14	112	5	4.46
G15	97	6	6.18
G16	121	7	5.78
G17	110	8	7.27
G18	117	6	5.12
G19	121	8	6.6
G20	121	8	6.6

**Table 5:** Number of larvae with melanotic tumors observed in *hml-GAL4>DNMT3L* flies across various generations.

Generation No.	Total larvaescreened	Larvae withtumor	% of larvaewith tumor
G5	118	8	6.7
G6	121	7	5.78
G7	97	5	5.1
G8	103	6	5.8

**Table 6:** Number of flies with rough eye phenotype observed in *GMR-GAL4>DNMT3L* flies across various generations.

Generation No.	Total fliesscreened	Flies withRough Eye	% of flies withRough Eye
G5	123	123	100
G6	132	132	100
G7	103	103	100
G8	117	117	100
G9	118	118	100
G10	107	107	100

**Table 7:** H3K4me_3_ association with promoters (+/−1000 bp from TSS) of misregulated genes.

	Total		Upregulated		Down regulated	
	No. of genepromoters associatedwith H3K4me_3_	No. of genepromoters devoidof H3K4me_3_	No. of genepromoters associatedwith H3K4me_3_	No. of genepromoters devoidof H3K4me_3_	No. of genepromoters associatedwith H3K4me_3_	No. of genepromoters devoidof H3K4me_3_
G1	26.09	73.91	17.48	82.52	34.62	65.38
G2	30.53	69.47	35.67	64.33	29.75	70.25
G4	40.09	59.91	45.75	54.25	35.34	64.66
G5	43.05	56.95	47.03	52.97	42.59	57.41
G5Phen	63.30	36.70	47.42	52.58	77.88	22.12
Total gene set	53.27	46.73				

*Drosophila* modENCODE data (Comprehensive encyclopedia of genomic functional elements in the model organisms *C. elegans* and *D. melanogaster*; http://www.modencode.org/; date of access, 15/9/15) for histone modifications (for 3^rd^ instar larval stage) was extracted for the promoters of the misregulated genes and used for analysis.

**Table 8:** Involvement of piwi protein in the inheritance of epimutations in *DNMT3L* expressing flies.

Crosses	No. of larvae with tumors/ Total No. of Larvae (%)			
	Set 1	Set 2	Set 3	Average (%)
G5(*Actin-3L*) X G5(*Actin-3L*)	6/88(6.8)	7/96 (7.3)	5/87 (5.7)	6.6
G5 (*Actin-3L*) X *piwi*	0/103 (0)	0/103 (0)	0/106 (5)	0
*piwi-Act-GAL4* X *piwi-Act-GAL4*; G1	0/116 (0)	0/107 (0)	0/102 (0)	0

*piwi* – flies mutant for piwi protein (*piwi*^*06843/*^^*CyO*^*; ry*^*506/+*^); *Actin-3L* - DNMT3L expressing flies with *Actin-GAL4* driver; *piwi-Act-GAL4*: DNMT3L expressing *Actin-3L* flies with mutant *piwi* gene.

**Table 9:** Maternal inheritance of epimutations.

Crosses	No. of larvae with tumors/Total No. of Larvae (%)			
	Set 1	Set 2	Set 3	Average (%)
G1 ♀ X G1 ♂	0/120 (0)	0/115 (0)	0/131 (0)	0
G5 ♀ X G5 ♂	6/112 (5.3)	7/102 (6.9)	6/121 (5)	5.7
G1 ♀ X G5 ♂	0/110 (0)	0/115 (0)	0/98 (0)	0
G5 ♀ X G1 ♂	3/104 (2.9)	4/108 (3.7)	4/111 (3.6)	3.4

G1 and G5 – *Tub-3L* flies from 1^st^ and 5^th^ generation respectively.
