# Author Correction: DNMT3L enables accumulation and inheritance of epimutations in transgenic *Drosophila*

**DOI:** 10.1038/s41598-022-08888-5

**Published:** 2022-03-21

**Authors:** Amitava Basu, Archana Tomar, Vasanthi Dasari, Rakesh Kumar Mishra, Sanjeev Khosla

**Affiliations:** 1 Centre for DNA Fingerprinting and Diagnostics (CDFD), Hyderabad, India; 2 Centre for Cellular and Molecular Biology (CCMB), Council of Scientific and Industrial Research (CSIR), Hyderabad, India; 3 Graduate Studies, Manipal University, Manipal, India

Correction to: *Scientific Reports* 10.1038/srep19572, published online 22 January 2016

This Article contains an error in Figure 8C. The image displaying an example of the Western blot of H3K9me3 was captured in the wrong orientation (upside down), and is displayed at 180 degree rotation with respect to the other blots in the panel.

A new figure has been compiled to display data from a biological replicate conducted at the same time as the original experiment and is shown below as Figure 1.

**Figure 1:**
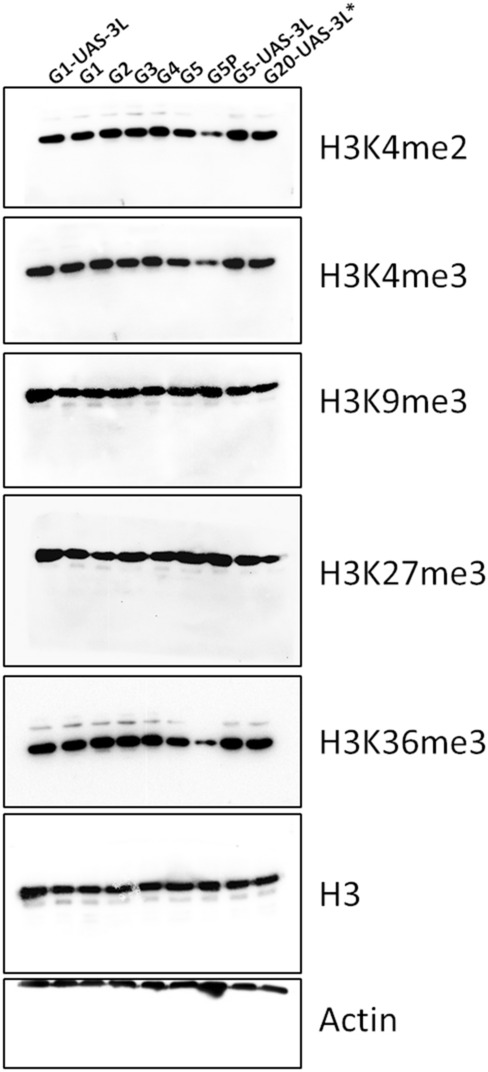
Western blot analysis for the various histone modification as indicated, performed on larvae from the various generations of control and DNMT3L expressing Drosophila larvae. G1-UAS-3L and G5-UAS-3L are control larvae without GAL4 driver from G1 and G5 generation respectively. G1 to G5-Tub-3L larvae from the indicated generation. G5P-G5 Tub-3L larvae that had melanotic tumors. G20-UAS-3L* denotes larvae from G20 generation after crossing out of the Tubulin-GAL4 driver. Actin was used as a loading control.

Similarities in presentation of the H3K9me3 and H3 blots arise from the use of a stripping and re-probing protocol as part of the Western blotting method, which was not described in the Article and which is included below.

**Western blotting, probe stripping and reprobing**

25 µg of lysate in loading buffer (For 7.5 ml of 4X buffer—1.5 mL 1 M Tris–HCl [pH 6.5], 3 mL of 1 M DTT, 0.6 g of SDS, 30 mg of Bromophenol Blue, 2.4 mL of Glycerol—volume made up to 7.5 mL with water) was resolved on a 12% SDS gel in running buffer (25 mM Tris base, 190 mM glycine, 0.1% SDS—pH adjusted to 8.3) and then transferred onto a PVDF membrane in transfer buffer (25 mM Tris base, 190 mM glycine, 20% methanol—pH adjusted to 8.3). The membrane was blocked with 5% skimmed milk in TBST (Tris [pH 7.5] 20 mM, NaCl 150 mM and 0.1% Tween 20) for 1 h and incubated with primary antibody (at 1:5000 dilution) overnight at 4 °C followed by 3X TBST washes for 5 min. The blot was then incubated with HRP conjugated secondary antibody for 1 h at 1:5000 dilution followed by 3X TBST washes for 5 min. The blots were developed with western blot substrate and images captured in a Chemidoc.

For reprobing a blot with different antibody, the western blots after developing for a particular antibody was washed 3 times with TBST for 10 min at RT. The blot was treated with stripping buffer (62.5 mM Tris HCl (pH6.8), 2%SDS, 100 mM β-ME) at 50 °C for 30 min with gentle shaking. The stripping buffer was washed off with TBST.

After this the blot was again blocked with 5% skimmed milk in TBST, washed and probed with different primary antibody as described above.

These changes do not affect the conclusions of the Article.

