# Supplementary material for: DNMT3L enables accumulation and inheritance of epimutations in transgenic Drosophila
Source: Sci Rep. 2016 Jan 22;6:19572. doi: 10.1038/srep19572 (PMC4726149; doi:10.1038/srep19572)
Supplement: Supplementary Information [file srep19572-s1.pdf]

DNMT3L enables accumulation and inheritance of epimutations in transgenic *Drosophila*

Amitava Basu[1,3], Archana Tomar[1], Vasanthi Dasari[2], Rakesh Kumar Mishra[2*], Sanjeev Khosla[1*]

[1] Centre for DNA Fingerprinting and Diagnostics (CDFD), Hyderabad, India; [2] Centre for Cellular and Molecular Biology (CCMB), Council of Scientific and Industrial Research (CSIR), Hyderabad, India; [3]Graduate Studies, Manipal University, Manipal, India.

* Correspondence to:
Dr. Sanjeev Khosla,CDFD, Tuljaguda (Opp. MJ Market), Nampally, Hyderabad-500001, India
Tel. 0091-40-24749410; Fax. 0091-40-24749448; Email: sanjuk@cdfd.org.in

Dr. Rakesh Kumar Mishra, Centre for Cellular and Molecular Biology (CCMB), Council of Scientific and Industrial Research (CSIR), Hyderabad, India. Tel. 0091-40-27192658; Fax. 0091-40-27190591; Email: mishra@ccmb.res.in

Supplementary Information

Data for the transcriptional profiling of transgenic DNMT3L larvae from the different generations performed using Affymetrix *Drosophila* Gene 1.1 ST array has been submitted to Gene Expression Omnibus (GEO), NCBI at
http://www.ncbi.nlm.nih.gov/geo/query/acc.cgi?acc=GSE68861
and is scheduled to be released on Dec 31, 2015.

Supplementary Table S1

LINE	***CHROMOSOME***	***EYE* color (male)**	***EYE* color (female)**	***MARKER***
6.6.2	3	Dark red	Orange	Tm2
31.1	1	Red	Orange	Fm3/Fm7a
38.1.1	2	Dark Red	Red	CyO
38.2.2	3	Orange	Light Orange	Tm6
42.1.2	3	Red	Orange	Tm2
44.1.1	3	Orange	Yellow	Tm2

S1: Description of transgenic *DNMT3L* Drosophila lines. *DNMT3L* transgene under the control of an *hsp70* promoter that contained *GAL4* binding sites (UAS) was generated by P-element mediated

germline transformation. The eye color of the transgenic flies was due to the presence of *mini-white* reporter gene.

Supplementary Table S2

	**Transgenic flies with wing phenotype (%)**		
**LINE**	***Tub-3L***	***Actin-3L***	***da-3L***
**6.6.2**	50	55.3	54.5
**31.1**	100	67.3	71.9
**38.1.1**	61.4	60.5	54.8
**38.2.2**	45.5	50	54.6
**42.1.2**	65.3	70.7	55.3
**44.1.1**	52.7	66	67.2

S2: Number (%) of transgenic Drosophila expressing DNMT3L with wing phenotype. *Tub-3L*, *Actin-3L*, *da-3L* - transgenic DNMT3L flies with *Tubulin*, *Actin* or *daughterless GAL4* driver respectively.

Supplementary Table S3

**Generation**	**No. of upregulated genes**	**No. of down regulated genes**	**Total misregulated genes**
**G1**	103	102	205
**G2**	157	354	511
**G4**	495	887	1382
**G5**	1129	1744	2873
**G5P**	1588	2142	3730
**G20***	57	17	74

S3: Number of misregulated genes based on microarray analysis. The number of misregulated genes in the indicated generation for Tub-3L larvae were calculated in comparison with UAS-3L flies in generation 1 (see flow chart in supplementary figure S3). *Tub-3L*, - transgenic DNMT3L flies with *Tubulin-GAL4* driver. UAS-3L- Transgenic DNMT3L flies without *GAL4* driver.

Supp. Figure S1

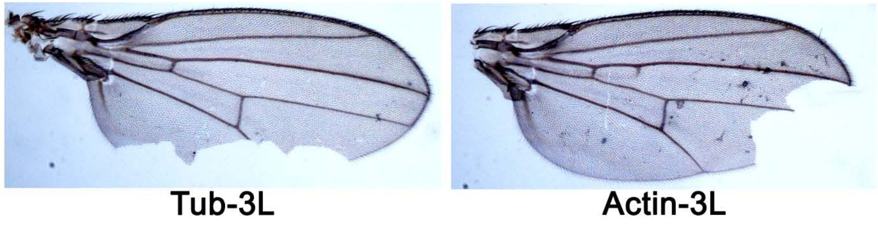

Supp. Figure S2

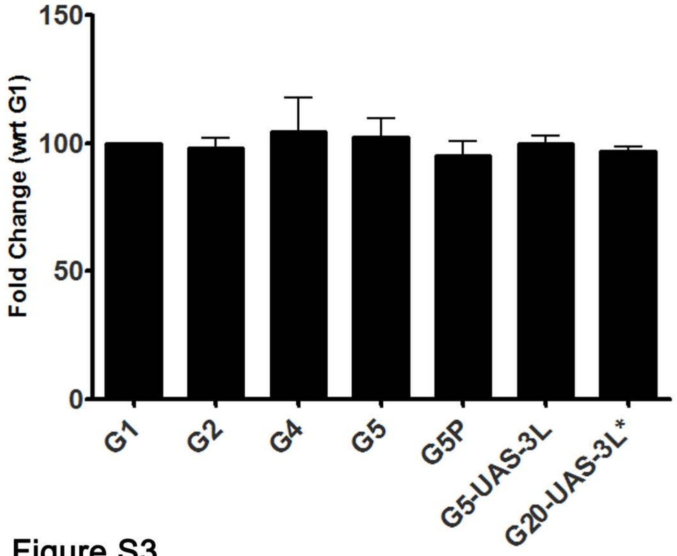

Supp. Figure S3

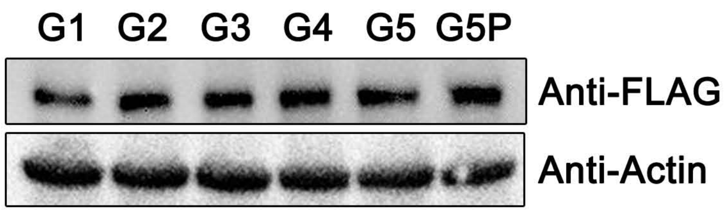

Supp. Figure S4

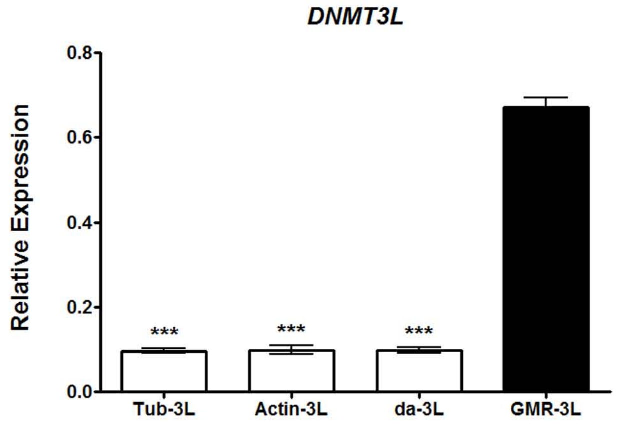

Supp. Figure S5

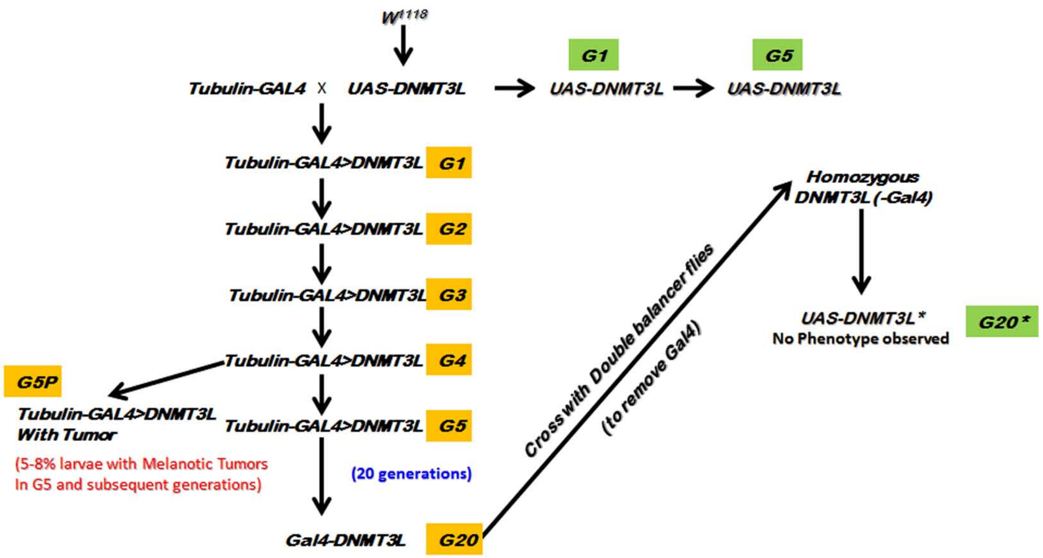

Supp. Figure S6

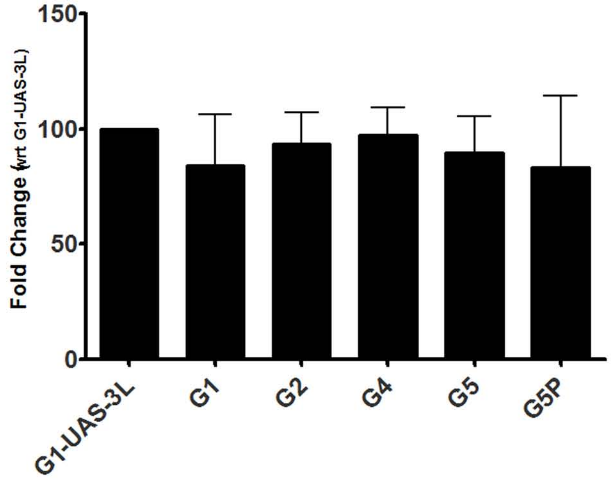

Supp. Figure S7

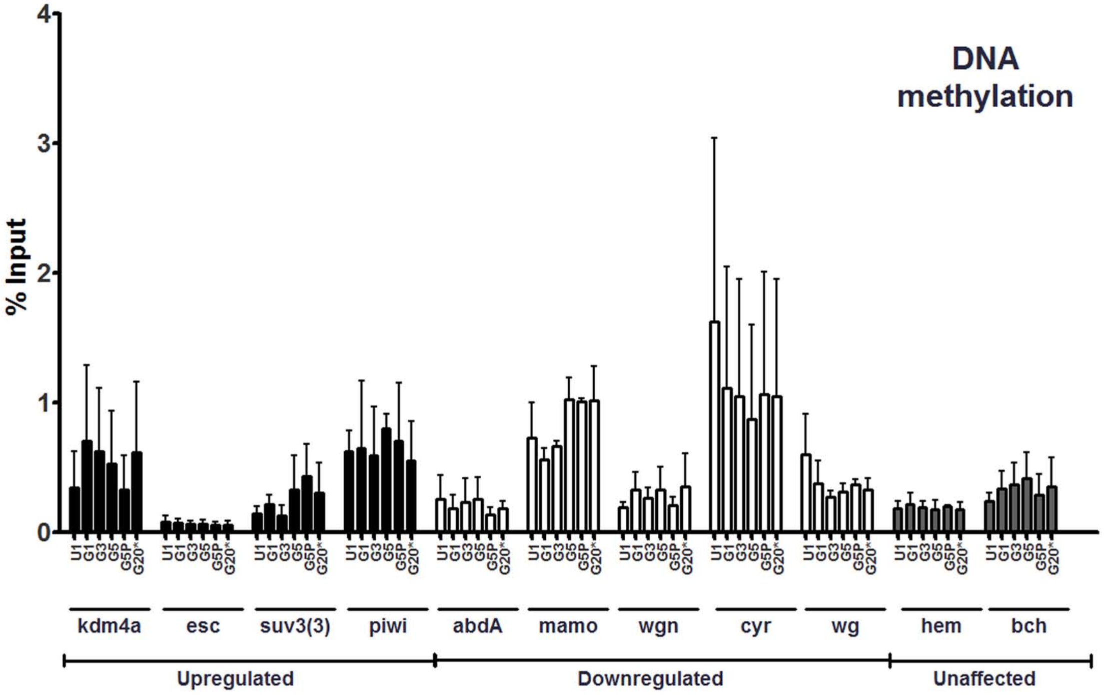

Supp. Figure S8

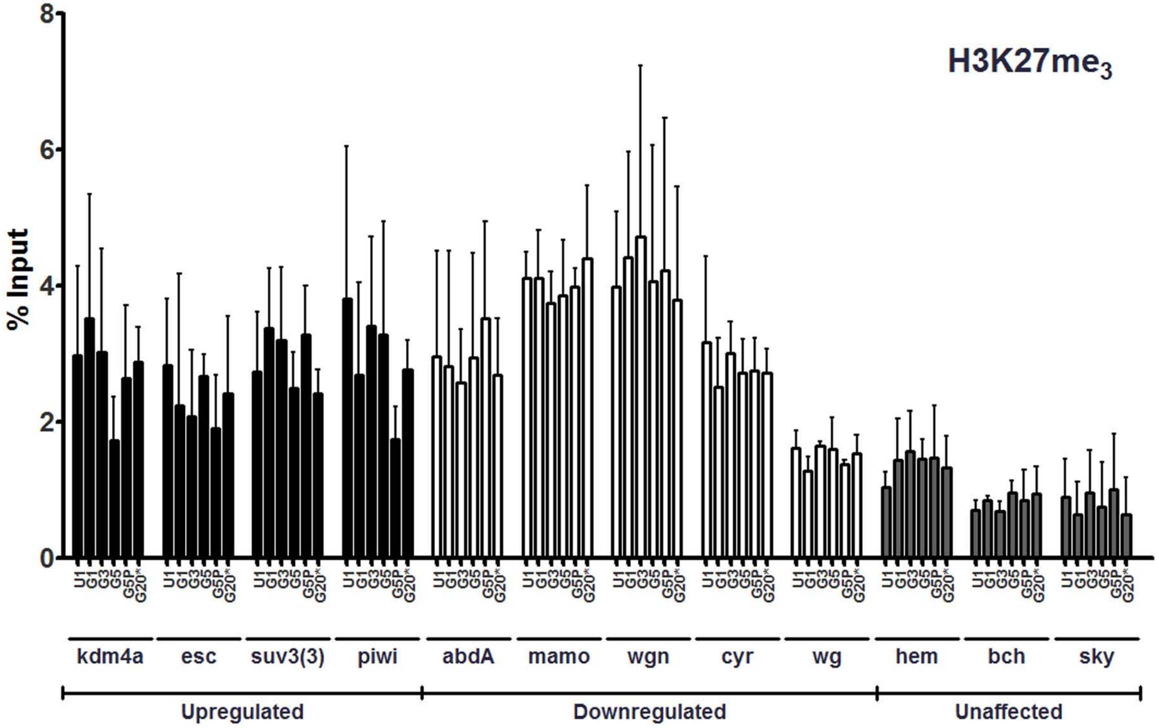

Supp. Figure S9

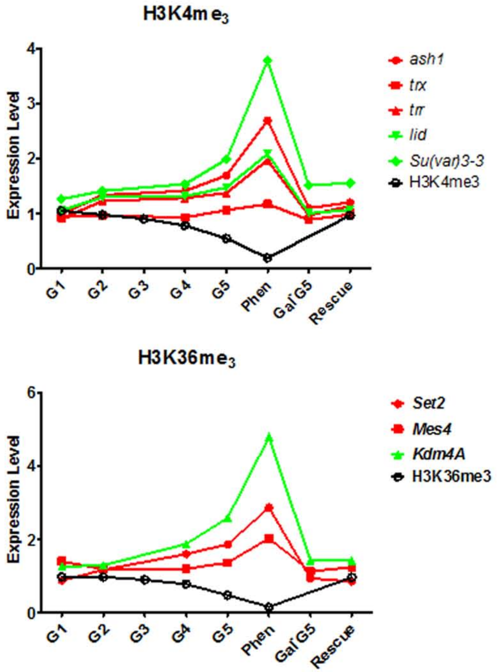

Supplementary Figure Legends:

Supp Figure S1: Ectopic *DNMT3L* expression causes broken wing phenotype in the transgenic *Drosophila*. Transgenic DNMT3L flies showing broken wings in the DNMT3L expressing flies. *Tub-3L, Actin-3L* - transgenic DNMT3L flies with *Tubulin* or *Actin GAL4* driver respectively.

Supp Figure S2: *DNMT3L* expression does not change across the various generations. The expression of DNMT3L was examined by Qualitative RT-PCR. rp49 expression was used as a control. The values are plotted as fold change with respect to DNMT3L expression in G1, *Tub-3L* 3rd instar larvae. G1, G2, G4, G5, denotes *Tub-3L* larvae from the particular generation. G5P denotes 5th generation *Tub-3L* larvae with melanotic tumors. *G5-UAS-3L* - 5th generation transgenic *UAS-DNMT3L* flies (without any *GAL4* driver). G20-*UAS-3L**- denotes G20 flies after removal of the *GAL4* driver by crossing flies with double balancers.

Supp Figure S3: *DNMT3L* expression does not change across the various generations. Protein expression of DNMT3L was examined by Western blotting. Anti-FLAG antibody was used to detect DNMT3L that had a FLAG tag. Actin was used as the loading control. G1, G2, G4, G5, denotes *Tub-3L* larvae from the particular generation. G5P denotes 5th generation *Tub-3L* larvae with melanotic tumors.

Supp Figure S4: Comparison of *DNMT3L* expression in the eyes of *Tub-3L, Actin-3L, da-3L* and *GMR-3L* flies. The expression of DNMT3L was examined by Qualitative RT-PCR. rp49 expression was used as a control. *Tub-3L*, Actin-3L, da-3L, *GMR-3L*: 5th generation transgenic flies expressing *DNMT3L* with *Tubulin, Actin, daughterless* or *GMR-GAL4* driver. The error bars

represent Standard Deviation (S.D.). * indicate significant difference (Student's t test, *** - $p < 0.001$).

Supp Figure S5: Flow chart showing the various crosses that were performed in the study. G1-G5, G20 – Generation numbers. Green– UAS-DNMT3L larvae; Yellow – Tub-3L larvae, G5P denotes 5th generation *Tub-3L* larvae with melanotic tumors. *UAS-3L*.

Supp Figure S6: DNA methylation levels do not change across various generations. The DNA methylation level was examined by MeDIP on adaptor ligated sonicated Drosophila DNA followed by quantitative Real-Time PCR using adaptor primers. rp49 expression was used as a control. The values are plotted as fold change with respect to DNA methylation in G1 UAS-3L larvae. G1, G2, G4, G5, denotes *Tub-3L* larvae from the particular generation. G5P denotes 5th generation *Tub-3L* larvae with melanotic tumors. *G1-UAS-3L*, - 1st generation transgenic *UAS-DNMT3L* flies (without any *GAL4* driver).

Supp Figure S7: DNA methylation levels do not change across various generations at specific gene loci. The DNA methylation level was examined by MeDIP followed by quantitative Real-Time PCR using primers for specific gene promoters as indicated below the X-Axis. rp49 expression was used as a control. The values are plotted as fold change with respect to DNA methylation in G1 UAS-3L larvae. U1- control larvae without GAL4 driver from the 1st generation G1, G3 and G5- *Tub-3L* larvae from the indicated generation. G5P denotes 5th generation *Tub-3L* larvae having melanotic tumors. G20* denotes larvae from G20 generation after crossing out of the *Tubulin-GAL4* driver.

Supp Figure S8: ChIP analysis for association of $H3K27me_3$ with specific gene promoters. Graphical representation of the association of $H3K27me_3$ with the promoter of selected

upregulated, downregulated and unaffected genes tested by ChIP using specific antibody. Results are represented as % input. The names of the specific gene promoters tested are given below the X-axis. U1- control larvae without GAL4 driver from the 1st generation G1, G3 and G5- *Tub-3L* larvae from the indicated generation. G5P denotes 5th generation *Tub-3L* larvae having melanotic tumors. G20* denotes larvae from G20 generation after crossing out of the *Tubulin-GAL4* driver. The error bars represent Standard Deviation (S.D.). * indicate significant difference (Student's t test, * - $p < 0.05$, ** - $p < 0.01$).

Supp Figure S9: Change in the H3K4 and H3K36 methylation levels correlate with the expression level of Histone demethylases Graphical representation of gene expression changes (shown as fold change with respect to UAS-3L, G1) for the depicted genes (see legend) are plotted along with the genome-wide level of $H3K4me_3$ (upper panel) and $H3K36me_3$ levels (lower panel) across the various generations. G1, G2, G4, G5, denotes *Tub-3L* larvae from the particular generation. G5P denotes 5th generation *Tub-3L* larvae with melanotic tumors. *G5-UAS-3L*, - 5th generation transgenic *UAS-DNMT3L* flies (without any *GAL4* driver). G20* - denotes larvae from G20 generation after crossing out of the *Tubulin-GAL4* driver.

ChIP PCR primers

kdm4aF	5’ GTGCAGCCAAATCCGAAGAA 3’
kdm4aR	5’ TCAGGTACAGACGCAATGGT 3’
escF	5’ CTTCCCAGCCCGATGAAGTA 3’
escR	5’ CAGCTCGTTAATGGCCTGTC 3’
su(var)3-3F	5’ GATGGCCGGGACTAAACAAA 3’
su(var)3-3R	5’ AAAACGCTTCCCTCTGCTTG 3’
piwiF	5’ GGACCCGGCTAGTGATACTC 3’
piwiR	5’ TTTGTAGTATGCTTTGCCTGC 3’
abd-AF	5’ CTCTCCGTTCCAGACTTCGA 3’
abd-AR	5’ GTGAGTTTCCTGCGGCTTTT 3’
mamoF	5’ GCTCTCTTTTGGGCCGAAAA 3’
mamoR	5’ GAAAGAGCGAAAAGGCCGAA 3’
wgnF	5’ TTCAGCTCCCATCTCCGATC 3’
wgnR	5’ CCCAGCTCCACGAAATGC 3’
cyrF	5’ TATGTGCATTGCGGTTGAGG 3’
cyrR	5’ GCACGGACTGAATCACTACG 3’
wgF	5’ TGTTCTTACTCACTCGCCGT 3’
wgR	5’ GCGCTGGAAAAGAGTTAGGG 3’
hemF	5’ CCGGCCATTCTTCTTAAGGC 3’
hemR	5’ TTGACTTCCGGGTACTGTCC 3’
bchF	5’ GCCGACTCTCTGCAGATTTG 3’
bchR	5’ TGCAAATTTTAACGCCAGGGA 3’
skyF	5’ TATCCAAAACCAGGGGCACA 3’
skyR	5’ TAGGGAGAGAGCAGCAAGTG 3’
kdmExF1	5' AAACTCCCAACCATTGCGTC 3'
kdmExR1	5' GGCAGTTCATTCCATAGGCG 3'
escExF1	5' TGGAAACCGGGACAACTACA 3'
escExR1	5' GAAGCCGAATCTCACGAACC 3'
suv3ExF1	5' GAAATGTTGTCAAGCTGCGC 3'
suv3ExR1	5' CGTAGAGTCGGCAGTGTTTG 3'
piwiExF1	5' CTGCCCGAGAGATACGACTT 3'
piwiExR1	5' GTGAGAGACCCATGCTGCTA 3'
abdAExF1	5' GGACAAGAGCAATCACGACC 3'
abdAExR1	5' GTGGAAGATTGGCTCGTTGG 3'
mamoExF1	5' TAATTTCAATCCGCAGCCCC 3'
mamoExR1	5' GCTGTCTTGATTCCCTCTCG 3'
wgnExF1	5' AAGCGAGAATGGATCCGGAA 3'
wgnExR1	5' CTTCCTCCGTCCTCCTCCTA 3'
cyrExF1	5' GTTCTCATCCAGCAGCGAAT 3'
cyrExR1	5' TCCGCCACGCAATCAATTAG 3'
wgExF1	5' CAAGCTGTGTCGGACCAAAA 3'
wgExR1	5' TACAAAGAACGAAGAGGGCG 3'
hemExF1	5' CAAAACTGGCCCGTAACGAA 3'
hemExR1	5' AACAGGGCTCCGAAGATGTT 3'
bchExF1	5' AAGACAAACTAAGCACCGCC 3'
bchExR1	5' CAAAAGGTGCGGCGATATCC 3'

Quantitative real time PCR primer for Dnmt3L were

Dnmt3LF 5’ CTTCGATCTTGTGTACGGCG 3’

Dnmt3LR 5’ GGTCTTCCTTGTTCAGCACC 3’

Sequence of the primers used for adapter ligation were

LK102 5’ GCGGTGACCCGGGAGATCTGAATTC 3’

LK103 5’ GAATTCAGATC 3’
